# Usefulness of a Hepatitis B Surface Antigen-Based Model for the Prediction of Functional Cure in Patients with Chronic Hepatitis B Virus Infection Treated with Nucleos(t)ide Analogues: A Real-World Study

**DOI:** 10.3390/jcm10153308

**Published:** 2021-07-27

**Authors:** Gian Paolo Caviglia, Yulia Troshina, Enrico Garro, Marcantonio Gesualdo, Serena Aneli, Giovanni Birolo, Fabrizia Pittaluga, Rossana Cavallo, Giorgio Maria Saracco, Alessia Ciancio

**Affiliations:** 1 Department of Medical Sciences, University of Torino, 10123 Turin, Italy; yulia.troshina@unito.it (Y.T.); enrico.garro@edu.unito.it (E.G.); marcantonio.gesualdo@unito.it (M.G.); serena.aneli@unito.it (S.A.); giovanni.birolo@unito.it (G.B.); giorgiomaria.saracco@unito.it (G.M.S.); 2 Department of Biology, University of Padua, 35122 Padova, Italy; 3 Microbiology Unit, A.O.U. Città della Salute e della Scienza, 10126 Torino, Italy; fpittaluga@cittadellasalute.to.it (F.P.); rossana.cavallo@unito.it (R.C.); 4 Gastroenterology Unit, A.O.U. Città della Salute e della Scienza, 10126 Torino, Italy

**Keywords:** antiviral therapy, biomarker, chronic liver disease, HBsAg, HBV

## Abstract

In patients with chronic hepatitis B (CHB) under long-term treatment with nucleso(t)ide analogues (NAs), the loss of hepatitis B surface antigen (HBsAg) is a rare event. A growing body of evidence supports the use of quantitative HBsAg for the prediction of functional cure, although these results are mainly derived from studies performed on Asian patients with hepatitis B e antigen (HBeAg)-positive CHB. Here, we investigated the clinical role of quantitative HBsAg in a real-life cohort of CHB patients under treatment with NAs in a tertiary care center from North-West Italy. A total of 101 CHB patients (HBeAg-negative, *n* = 86) undergoing NAs treatment were retrospectively enrolled. HBsAg was measured at baseline (T0), 6 months (T1), 12 months (T2) and at the last follow-up (FU). Median FU was 5.5 (3.2–8.3) years; at the end of FU, 11 patients lost the HBsAg (annual incidence rate = 1.8%). Baseline HBsAg levels were significantly different between patients with no HBsAg loss and those achieving a functional cure (3.46, 2.91–3.97 vs. 1.11, 0.45–1.98 Log IU/mL, *p* < 0.001). Similarly, the HBsAg decline (Δ) from T0 to T2 was significantly different between the two groups of patients (0.05, −0.04–0.13, vs. 0.38, 0.11–0.80 Log IU/mL, *p* = 0.002). By stratified cross-validation analysis, the combination of baseline HBsAg and ΔHBsAg T0–T2 showed an excellent accuracy for the prediction of HBsAg loss (C statistic = 0.966). These results corroborate the usefulness of quantitative HBsAg in Caucasian CHB patients treated with antivirals for the prediction of HBsAg seroclearance.

## 1. Introduction

Hepatitis B virus (HBV) infection is a major health problem [1]. Globally, 257 million people are chronically infected with the virus (estimated prevalence: 3.7%) [2]. However, the epidemiological scenario varies greatly across different geographic regions, mainly due to different socioeconomic conditions and an uneven vaccination coverage [3,4]. In Italy, the prevalence of chronic HBV infection progressively declined in native Italians since the implementation of compulsory vaccination in 1991 [5]. It has then remained stable due to the input of new infections brought by HBV-infected immigrants [6,7]. To date, the clinical presentation of CHB shifted toward older ages and more severe diseases [8].

Therapeutic strategies for CHB include finite treatment with pegylated-interferon (PEG-IFN) and indefinite treatment with nucleos(t)ide analogues (NAs) [9]; the latter allows an effective suppression of viral replication, normalization of alanine aminotransferase (ALT) and thus the prevention of liver disease progression [10]. Despite the low cumulative rate of hepatitis B surface antigen (HBsAg) loss (i.e., functional cure), international guidelines recommend long-term administration of NAs with a high genetic barrier to resistance regardless of liver disease severity [11]. Consistently, previous Italian series showed that NAs therapy was the treatment of choice, not only in IFN-experienced CHB patients but also as first-line approach [12,13,14].

In the last decade, the measurement of serum HBsAg in patients undergoing NAs treatment gained growing relevance for the prediction of HBsAg clearance [15]. Low pre-treatment HBsAg levels were significantly associated with HBsAg loss, particularly in hepatitis B e antigen (HBeAg)-positive patients [16]. Furthermore, several pieces of evidence suggest that on-treatment HBsAg decline was able to predict HBsAg seroclearance, both in HBeAg–positive and –negative patients [17,18,19]. However, these results predominantly derive from studies involving Asian patients infected with genotypes B and C. To date, in HBeAg–negative Caucasian patients under long-term treatment with NAs, the predictive value of HBsAg kinetic is less clear [20,21].

The aim of the present study was to investigate the clinical role of quantitative HBsAg in a real-life cohort of CHB patients under treatment with NAs in a tertiary care center from North-West Italy.

## 2. Materials and Methods

### 2.1. Patients

This observational study included patients with CHB that underwent treatment with NAs retrospectively recruited at the outpatient clinic of the Unit of Gastroenterology of Città della Salute e della Scienza di Torino–Molinette Hospital, Turin, Italy, between November 2011 and June 2020.

The inclusion criteria included age of ≥18 years, HBsAg–positivity for at least 6 months and having received at least 18 months of consecutive NAs treatment. An additional inclusion criterion was the availability of HBsAg measurement during NAs administration according to the following minimum schedule: baseline (T0), 6th month (T1), 12th month (T2) and last follow-up (FU). No restriction was set concerning previous NAs or IFN-based treatment. Patients were censored in case of death, loss to FU and HBsAg clearance. We excluded patients co-infected with hepatitis C virus or hepatitis D virus (HDV) and those with human immunodeficiency virus infection, patients with a diagnosis of hepatocellular carcinoma, patients receiving NAs as prophylaxis for the risk of HBV reactivation and those with no signed informed consent.

### 2.2. Study Endpoint

The endpoint of the study was the comparison of baseline HBsAg values and HBsAg kinetics between patients that achieved a functional cure during NAs treatment and those still HBsAg-positive at the last FU.

### 2.3. Definitions

Functional cure was defined as HBsAg loss, with or without anti-HBs seroconversion. Virologic response was defined as the sustained suppression of HBV DNA to undetectable levels. Biochemical response was defined as the sustained ALT normalization (upper limit of normality, ULN = 40 IU/L) [11].

### 2.4. Data Collection

A specific database was prepared for the collection of demographic, biochemical, virologic and clinical variables relevant to the study. As per the standard of care, all patients underwent a periodical liver ultrasound examination and esophagogastroduodenoscopy according to their stage of liver disease. The presence of cirrhosis was assessed by liver biopsy, liver elastography (FibroScan^®^, Echosens™, Paris, France) or by hepatic ultrasound features and endoscopic signs of portal hypertension [11,22].

### 2.5. Serology and Virology

All the serologic and virologic diagnostics were performed at the centralized reference laboratory of Molinette Hospital. In particular, the ARCHITECT-QT assay (Abbott Diagnostics, Abbott Park, IL, USA) was used for the measurement of HBsAg in serum [23]. Plasma HBV DNA was detected and quantified with the COBAS/AmpliPrepCOBAS TaqMan HBV assay, version 2.0 (Roche Molecular Diagnostics, Branchburg, NJ, USA) [24].

### 2.6. Statistical Analysis

Data were reported using the median and interquartile range (IQR) or the number and percentage for continuous and categorial variables, respectively. Data normality was checked by the D’Agostino-Pearson test. Comparison between unpaired groups was performed by the Mann-Whitney test for continuous variables and by the Fishers’ Exact test or chi-squared test for categorical variables. For paired analysis, we used the Wilcoxon or McNemar test for continuous or categorical data, respectively.

Predictiveness was evaluated by Harrell’s concordance index (C-index) and the area under the curve (AUC) of the receiver operating characteristic (ROC) curve. The ROC curve was computed considering events that took place within 10 years and individuals were censored after more than 10 years from the recruitment (10 and 13 individuals, respectively). Cut-off values with maximal Youden *J* statistics were selected from such curve.

Survival analysis was carried out according to the Kaplan-Meier method using the previously selected cut-off values; survival curves were compared using the log-rank test. Multivariate Cox regression analysis was used to evaluate the association between selected variables and the outcome; the strength of the association was reported as a hazard ratio (HR) and a 95% confidence interval (CI). C-index and AUC of the Cox model were estimated using cross-validation (5 splits and shuffling samples 20 times). Confidence intervals at a 95% confidence level have been estimated by bootstrapping 1000 times.

A two-tailed *p* value < 0.05 was considered statistically significant. All the statistical analyses were performed using MedCalc software, version 18.9.1 (MedCalc bvba, Os-tend, Belgium) and the Python packages scikit-learn (version 0.24.2) and scikit-survival (0.15.0).

## 3. Results

### 3.1. Characteristics of the Patients Included in the Study

The clinical records of 171 patients with CHB were screened. A total of 101 (59%) patients were included in the study. Twenty-nine patients were excluded due to chronic HDV infection (anti-HDV-positive/HDV RNA-positive), while 32 patients had a diagnosis of HBeAg–negative chronic infection (i.e., inactive carriers; persistent HBV DNA < 2000 UI/mL and ALT < 40 IU/mL) [11]. Another group of 9 patients had no indication of antiviral treatment due to intermediate HBV DNA levels (2000–20,000 IU/mL), persistently normal ALT and no or mild liver fibrosis (grey zone) [25]. The baseline characteristics of the patients enrolled are reported in Table 1.

The baseline median age was 56 (32–79) years and the male to female ratio was 69/32. Most patients were Caucasian (*n* = 98; 97%); 79 from Italy and 19 from East Europe. Nine (9%) patients were obese and 7 (7%) patients admitted alcohol abuse. Only 6 (6%) patients had type 2 diabetes requiring therapy while 29 (29%) had a diagnosis of hypertension. The principal risk factor for HBV infection was intrafamily exposure (*n* = 46; 46%), followed by hospitalization (*n* = 19; 19%) and sexual exposure (*n* = 5; 5%).

Only 18 (18%) patients had a diagnosis of cirrhosis. In one patient, the disease was complicated by ascites, while 6 (6%) patients showed esophageal varices at endoscopic examination. Consistently, biochemistry indicated an overall preserved liver function.

The majority of patients were anti-HBe-positive at baseline (*n* = 86; 85%); median HBsAg and HBV DNA levels were 3.25 (2.85–3.88) Log IU/mL and 3.45 (1.91–5.63) Log IU/mL, respectively. Forty-four (44%) patients were IFN-experienced while 61 (60%) patients reported previous NAs therapy. Median FU was 5.5 (3.2–8.3) years; 69 (68%) underwent antiviral treatment with entecavir (ETV), while 32 (32%) with tenofovir disoproxil fumarate (TDF). All the treated patients (100%) achieved virologic response, while 98 (97%) achieved biochemical response. At the end of FU, 11 patients achieved a functional cure (annual incidence rate = 1.8%). The loss of HBsAg was accompanied by seroconversion to anti-HBs in 3/11 (27%) patients (anti-HBs titers: 749 IU/mL, 138 IU/mL, and 124 IU/mL). No differences were observed in treatment duration between patients achieving a functional cure (7.5, 4.3–8.5 years) and those with no HBsAg loss (5.5, 2.9–8.2 years) (*p* = 0.433).

### 3.2. Comparison between Patients with or without HBsAg-Loss

**Figure 1:**
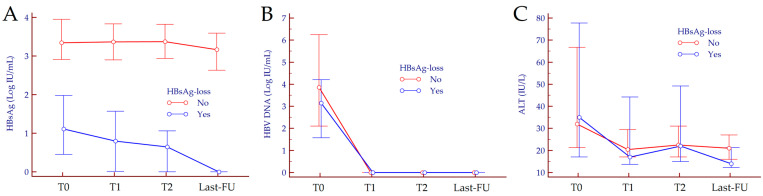
HBsAg (**A**), HBV DNA (**B**) and ALT (**C**) kinetics in patients achieving a functional cure and those with no HBsAg-loss. In both groups, HBsAg, HBV DNA, and ALT levels significantly declined from T0 to the last FU (Friedman test, *p* < 0.001). Data are depicted as the median and interquartile range. Abbreviations–alanine aminotransferase (ALT), follow-up (FU), hepatitis B surface antigen (HBsAg), hepatitis B virus (HBV), timepoint (T).

No significant differences were observed between patients achieving a functional cure and those without HBsAg loss regarding demographic and clinical characteristics (Table 1). At baseline, median HBsAg values were significantly lower in patients that achieved a functional cure compared to those still HBsAg-positive at the last FU (1.11, 0.45–1.98 Log IU/mL vs. 3.353, 2.91–3.95 Log IU/mL, *p* < 0.001). No differences were observed regarding circulating HBV DNA values and ALT levels (*p* = 0.130 and *p* = 0.870, respectively). We further analyzed the kinetics of HBsAg, HBV DNA and ALT from baseline to the last FU. Interestingly, we observed distinct differences according to the achievement of functional cure for HBsAg kinetics, but not for HBV DNA and ALT (Figure 1). HBsAg, HBV DNA and ALT values for each timepoint are reported in Table 2 (HBsAg values and kinetics are reported in absolute numbers in Supplementary Material Table S1).

**Figure 2:**
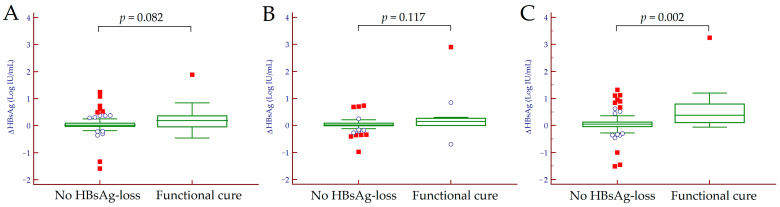
Comparison of HBsAg decline from baseline to T1 (**A**), from T1 to T2 (**B**) and from baseline to T2 (**C**) in patients achieving a functional cure and those with no HBsAg loss. Hollow circles indicate values that are larger than the upper quartile plus 1.5 times the interquartile range while red squares indicate values that are larger than the upper quartile plus 3 times the interquartile range. Abbreviations–HBsAg decline (ΔHBsAg), hepatitis B surface antigen (HBsAg).

Focusing on HBsAg, we calculated and compared the magnitude of HBsAg decline (ΔHBsAg) between patients achieving a functional cure and those with no HBsAg loss. We observed no differences between the magnitude of ΔHBsAg from baseline to T1 (*p* = 0.082) and from T1 to T2 (*p* = 0.117) between the two groups of patients, while we observed a significantly higher ΔHBsAg from baseline to T2 in patients achieving a functional cure (*p* = 0.002) (Figure 2). Median ΔHBsAg values in patients with or without HBsAg loss are reported in Table 3.

### 3.3. Prediction of HBsAg-Loss

**Figure 3:**
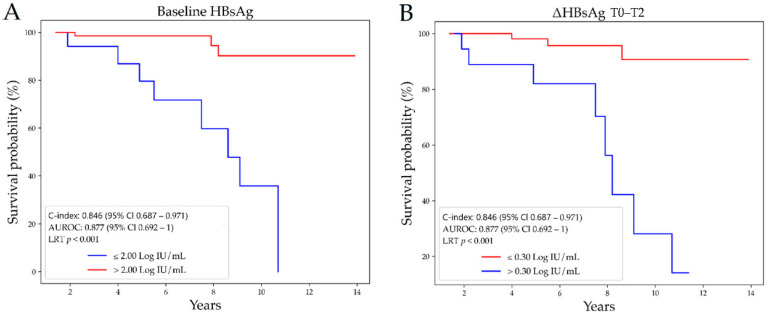
Survival curves for the prediction of HBsAg loss according to baseline HBsAg ≤ 2.00 Log IU/mL (**A**) and ΔHBsAg T0–T2 > 0.30 Log IU/mL (**B**). Survival curve analysis was performed according to the Kaplan–Meier method; the difference between the curves was assessed by a Log rank test. Abbreviations– area under the receiver operating characteristic curve (AUROC), HBsAg decline (ΔHBsAg), hepatitis B surface antigen (HBsAg), log-rank test (LRT), timepoint (T).

We performed a ROC curve analysis to investigate the performance of baseline HBsAg and ∆HBsAg T0–T2 to identify patients achieving functional cure in ten years. Baseline HBsAg showed a good diagnostic accuracy (AUC = 0.877, 95%CI 0.698–0.992) for the discrimination between patients that lost the HBsAg and those who did not; the optimal cut-off that maximized sensitivity (Se) and specificity (Sp) was ≤2.00 Log IU/mL. ∆HBsAg T0–T2 showed a moderate diagnostic accuracy (AUC = 0.818, 95%CI 0.589–1.000) for the identification of patients that lost the HBsAg; the optimal cut-off that maximized Se and Sp was >0.30 Log IU/mL. Differences between the two survival curves built from such selected cut-offs were significant (*p* < 0.001) (Figure 3). Accordingly, among patients that cleared the HBsAg, 9 out of 11 (82%) and 7 out of 11 (64%) had baseline HBsAg ≤ 2.00 Log IU/mL and ∆HBsAg T0–T2 values >0.30 Log IU/mL, respectively. Among patients still HBsAg-positive at the last FU, only 11 out of 90 (12%) had baseline HBsAg ≤ 2.00 Log IU/mL and 11 out of 90 (12%) had ∆HBsAg T0–T2 values >0.30 Log IU/mL. By multivariate Cox regression analysis, both baseline HBsAg and ∆HBsAg T0–T2 were significantly associated to HBsAg loss (HR = 0.20, 95%CI 0.09–0.44, *p* < 0.001, and HR = 9.40, 95%CI 3.29–26.82, *p* < 0.001, respectively). The prognostic indices obtained from the combination of both parameters were used to assess the discrimination ability of the predictive model; remarkably, we achieved C = 0.965, 95%CI 0.883–0.996.

The score of the multivariate model can be computed by the following formula:

−1.62 ∗ (baseline HBsAg Log IU/mL) + 2.24 ∗ (∆HBsAg T0–T2 Log IU/mL)

**Figure 4:**
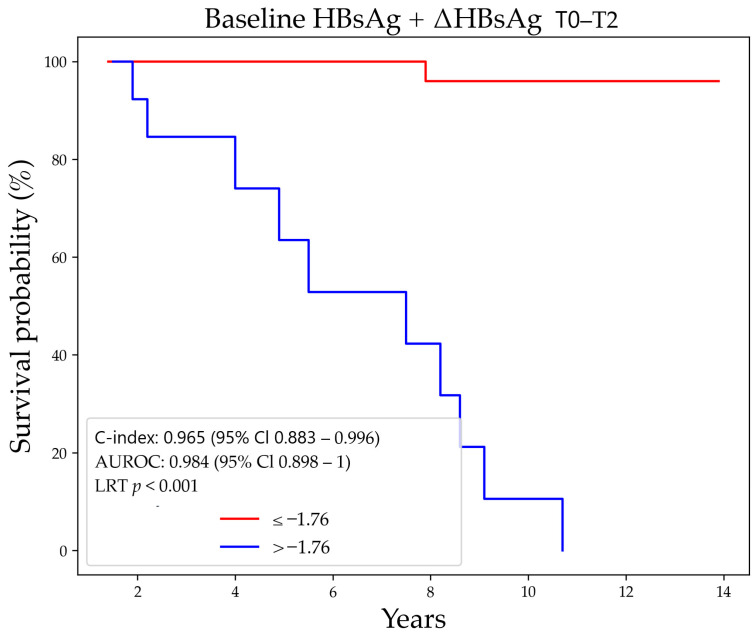
Predictiveness of the model combining baseline HBsAg ≤ 2.00 Log IU/mL and ΔHBsAg T0–T2 > 0.30 Log IU/mL for HBsAg loss. Abbreviations–HBsAg decline (ΔHBsAg), hepatitis B surface antigen (HBsAg), timepoint (T).

The optimal cut-off was −1.762, which yielded again significant different survival curves (*p* < 0.001) (Figure 4).

## 4. Discussion

The results of the present study showed that the measurement of baseline serum HBsAg and the magnitude of HBsAg decrease during treatment with third-generation NAs are useful to identify CHB patients with a higher likelihood of achieving functional cure. Our data obtained from a real-world clinical setting confirmed previous results mainly deriving from Asiatic cohorts of HBeAg-positive patients; we were able to confirm the usefulness of the HBsAg measurement in a cohort of CHB patients extremely heterogeneous in term of treatment duration, previous NAs and/or IFN-based treatment. Furthermore, we observed that the combination of both parameters (i.e., baseline HBsAg and HBsAg decline) was able to predict HBsAg seroclearance with high accuracy.

Overall, 11 out of 101 patients cleared the HBsAg during NAs therapy. In our study, the annual incidence rate of HBsAg loss was 1.8%; this result agrees with the estimated annual incidence of 1–2% reported both in Asian and in Western populations [26]. Previous studies showed that baseline HBsAg < 1000 IU/mL was the optimal cut-off for the prediction of HBsAg seroclearance (AUC = 0.860; negative predictive value (NPV) = 98%) in Chinese CHB patients (61.4% HBeAg-positive) undergoing lamivudine (LMV) treatment [27], while lower HBsAg levels after HBeAg seroclearance were associated with HBsAg loss in another Asiatic cohort of CHB patients, irrespectively of antiviral treatment [28]. In 390 Taiwanese HBeAg-positive CHB patients (genotype B and C) who had spontaneously cleared the HBeAg during FU, Tseng and colleagues observed that HBsAg serum levels <100 IU/mL at 1 year after HBeAg seroconversion, were able to predict HBsAg loss (HR = 24.3, 95%CI 8.7–67.5) within 6 years [29]. A recent European study investigated the changes of HBsAg titers in HBeAg-negative CHB patients undergoing low genetic barrier NAs and observed that lower baseline HBsAg levels were associated with on-therapy HBsAg drop <1000 IU/mL [30], while another Chinese study showed that low serum HBsAg level at year 1 of NAs treatment was an independent predictor of subsequent HBsAg <1000 IU/mL at year 8 of FU (HR = 0.24, *p* = 0.004) [31]. In the present study, we observed that baseline HBsAg values <2.00 Log IU/mL were significantly associated with HBsAg loss in a cohort of CHB patients undergoing ETV or TDF treatment, showing a good performance for seroclearance prediction (C = 0.846); this cut-off allowed us to correctly identify 88 out of 101 patients (accuracy = 87%).

Several studies showed that HBsAg decline is more pronounced in CHB patients treated with PEG-IFN compared to those treated with NAs [16,32]. For most CHB patients under NAs treatment, it has been estimated that a median HBsAg reduction of 0.08 Log IU/mL/year [33] is typical. Nonetheless, a conspicuous body of evidence supports the association between HBsAg decline and favorable therapeutic outcomes in patients under long-term NAs treatment [34]. In 7 out of 70 (10%) patients treated with LMV achieving HBsAg seroclearance, a greater HBsAg reduction (>0.166 Log IU/mL) hs been reported compared to the 63 patients still HBsAg-positive at the end of FU (AUC = 0.794; NPV = 98%) [27]. Another study performed on 266 HBeAg-positive CHB patients treated with TDF showed that an HBsAg decline ≥1.00 Log IU/mL at 6 months of therapy was independently associated to HBsAg loss (HR = 14.3, 95%CI 4.7–43.4) [35]. A more recent study including HBeAg-negative CHB patients, showed that an HBsAg decline > 0.3 Log IU/mL at 3 years of NAs treatment had Se = 100%, Sp = 81%, positive predictive value (PPV) = 42% and NPV = 100% for the identification of low HBsAg levels (< 120 IU/mL), and Se = 100%, Sp = 74%, PPV = 17% and NPV = 100% for the identification of HBsAg loss [20]. Finally, in a large cohort of 529 Asian CHB patients (195 HBeAg-positive and 334 HBeAg-negative) receiving ETV, it has been shown that an HBsAg decline ≥75% independently predicted HBsAg loss [18]. Furthermore, the authors reported that the combination of baseline HBsAg levels <3000 IU/mL and HBsAg decline ≥75% allowed to predict HBsAg seroclearance with PPV = 70% and NPV = 100% [18]. Finally, Jaroszewicz et al. showed that in CHB patients (mostly HBeAg-negative) undergoing treatments with NAs, HBsAg decrease during the first 6 months of NA therapy was not predictive for HBsAg loss, while a strong HBsAg decrease (>0.5 Log IU/mL) 2 years after HBV DNA suppression was associated with HBsAg loss [36]. Although we cannot firmly identify the optimal timing and the exact amount of HBsAg decrease during NAs, all these data highlight the importance of quantitative rather than qualitative HBsAg monitoring during treatment with NAs. Indeed, we observed that HBsAg decline >0.30 Log IU/mL was significantly associated with HBsAg loss (HR = 9.40, *p* < 0.001). Moreover, the model developed from the combination of baseline HBsAg values and ∆HBsAg T0–T2 used in our study demonstrates an excellent predictiveness for HBsAg loss (C = 0.965). These results further corroborate the usefulness of quantitative HBsAg monitoring in Caucasian CHB patients treated with antivirals; despite the low rate of HBsAg seroclearance during NAs therapy, the quantitative HBsAg qualifies as a reliable predictor of functional cure.

To note, the present research has some limitations including the retrospective design, and the clinical and virologic heterogeneity of the patients enrolled. Nonetheless, our results are in line with previous findings on this topic. Furthermore, the heterogeneity observed in our population resembles the real characteristics of CHB patients currently being referred to most of the tertiary care centers in Italy. Indeed, the majority of these patients have an HBeAg-negative serologic profile and have usually been exposed to previous PEG-IFN and/or NAs therapy. Here, we observed that HBeAg at baseline was not significantly different between patients experiencing HBsAg loss and those still positive at the last FU. However, the number of HBeAg-positive patients at baseline was quite low in our study. Considering that several studies reported that the rate of HBsAg decline is higher in HBeAg-positive vs. HBeAg-negative patients [37,38,39], we cannot exclude that the rate of HBsAg clearance is similar between HBeAg-negative and -positive CHB patients under NAs therapy. Another limitation may be found in the relatively low number of patients enrolled and the lack of a validation cohort. To overcome this issue, we applied a stratified cross-validation approach to assess the performance of the model; accordingly, the original sample was partitioned into a training set to train the model, and a test set to evaluate its performance, and the procedure was repeated multiple times. As a result, the model showed a high accuracy, with low risk of overfitting, and generalizability to independent datasets. Therefore, we believe that the results of the present study are robust and may be useful for the management of such patients, also in view of the arising concept of NAs cessation in HBsAg-positive patients [40].

## 5. Conclusions

In the present study, we showed that the measurement of baseline and on-treatment HBsAg decline are useful for the identification of CHB patients achieving a functional cure. Furthermore, the combination of both parameters allowed the prediction of HBsAg-loss with excellent accuracy. Further studies may investigate the applicability of the present findings for the definition of stopping rules for safely discontinuation of NAs therapy.

## Figures and Tables

**Table 1:** Baseline characteristics of the overall cohort of patients included in the study and according to HBsAg loss.

Characteristics	Overall	No HBsAg-Loss	Functional Cure	*p* Value
Patients, *n*	101	90	11	
Age (years), median (range)	56 (32–79)	57 (32–79)	56 (32–72)	0.624
Male gender, *n* (%)	69 (68%)	62 (69%)	7 (64%)	0.739
Nationality				
Italian, *n* (%)	79 (78%)	71 (79%)	8 (73%)	0.643
East Europe, *n* (%)	19 (19%)	16 (18%)	3 (27%)	
Other, *n* (%) ^A^	3 (3%)	3 (3%)	0	
Risck factors for HBV infection				
Family exposure, *n* (%)	46 (46%)	42 (47%)	4 (36%)	0.75
Sexual exposure, *n* (%)	5 (5%)	4 (4%)	1 (9%)	0.445
Hospitalization, *n* (%)	19 (19%)	15 (17%)	4 (36%)	0.212
Tattoo/Piercing, *n* (%)	1 (<1%)	1 (1%)	0	1
IVDU, *n* (%)	1 (<1%)	1 (1%)	0	1
Comorbidities				
Alcohol abuse, *n* (%) ^B^	7 (7%)	6 (7%)	1 (9%)	0.566
Obesity, *n* (%) ^C^	9 (9%)	9 (10%)	0	0.592
T2DM, *n* (%)	6 (6%)	5 (6%)	1 (9%)	0.09
Hypertension, *n* (%)	29 (29%)	27 (30%)	2 (18%)	0.505
Cirrhosis, *n* (%)	18 (18%)	15 (17%)	3 (27%)	0.408
Ascites, *n* (%)	1 (<1%)	1 (1%)	0	1
Esophageal varices, *n* (%)	6 (6%)	5 (6%)	1 (9%)	0.509
Serology				
HBsAg (Log IU/mL), median (IQR)	3.25 (2.85–3.88)	3.35 (2.91–3.95)	1.11 (0.45–1.98)	<0.001
HBV DNA (Log IU/mL), median (IQR)	3.45 (1.91–5.63)	3.86 (2.10–6.25)	3.15 (1.57–4.21)	0.13
HBeAg+/anti-HBe+	15/86	14/74	10-1	1
Biochemistry				
ALT (IU/L), median (IQR)	34 (21–68)	32 (21–67)	35 (17–78)	0.87
AST (IU/L), median (IQR)	29 (21–52)	30 (21–53)	24 (17–51)	0.249
γGT (IU/L), median (IQR)	24 (16–39)	24 (16–39)	19 (11–47)	0.33
ALP (IU/L), median (IQR)	65 (57–87)	65 (57–83)	81 (55–158)	0.519
Total bilirubin (mg/dL), median (IQR)	0.7 (0.6–0.9)	0.7 (0.5–0.9)	0.9 (0.7–1.3)	0.077
Albumin (g/L), median (IQR)	4.5 (4.2–4.7)	4.4 (4.1–4.7)	4.5 (4.3–4.7)	0.366
Platelet count (x10^9^/L), median (IQR)	181 (133–227)	185 (140–227)	154 (111–225)	0.235
Previous IFN treatment, *n* (%)	44 (44%)	41 (46%)	4 (36%)	0.75
Previous NAs treatment, *n* (%)	61 (60%)	53 (59%)	8 (73%)	0.519
Duration of previous NAs (years), median (IQR)	6 (3–10)	6 (3–10)	6 (1–12)	0.602

^A^ 2 patients were Asian, 1 was African and 1 was South American. ^B^ >140 g/week for woman and >210 g/week for men. ^C^ body mass index ≥ 30 kg/m^2^. Comparison between continuous variables was performed by the Mann-Whitney test. Comparison between categorical variables was performed by the Fisher’s exact test (dichotomous variables) or chi-squared test (non-dichotomous variables). Abbreviations–alkaline phosphatase (ALP), alanine aminotransferase (ALT), aspartate aminotransferase (AST), gamma-glutamyl transpeptidase (γGT), hepatitis B e antigen (HBeAg), hepatitis B surface antigen (HBsAg), hepatitis B virus (HBV), interferon (IFN), interquartile range (IQR), intravenous drug use (IVDU), number (*n*), nucleos(t)ide analogues (NAs), type 2 diabetes mellitus (T2DM).

**Table 2:** Comparison of HBsAg, HBV DNA and ALT levels according to HBsAg loss.

Biomarker	Timepoint	No HBsAg-Loss	Functional Cure	*p* Value
HBsAg (Log IU/mL)	T0	3.46 (2.91–3.97)	1.11 (0.45–1.98)	<0.001
	T1	3.39 (2.90–3.92)	0.80 (0.01–1.57)	<0.001
	T2	3.40 (2.95–3.89)	0.65 (0.01–1.06)	<0.001
	Last-FU	3.21 (2.63–3.78)	0 (0–0)	<0.001
HBV DNA (Log IU/mL)	T0	3.86 (2.10–6.25)	3.15 (1.57–4.21)	0.130
	T1	0 (0–0)	0 (0–0)	0.273
	T2	0 (0–0)	0 (0–0)	0.386
	Last-FU	0 (0–0)	0 (0–0)	1.000
ALT (IU/L)	T0	30 (20–67)	35 (17–78)	0.985
	T1	22 (17–31)	17 (14–44)	0.445
	T2	23 (17–31)	22 (15–49)	0.815
	Last-FU	21 (16–28)	14 (12–21)	0.038

*p* values were calculated by the Mann-Whitney test. Data are reported as the median and interquartile range. Abbreviations–alanine aminotransferase (ALT), follow-up (FU), hepatitis B virus (HBV), hepatitis B surface antigen (HBsAg), timepoint (T).

**Table 3:** Comparison of the magnitude of HBsAg decline between patients achieving a functional cure and those with no HBsAg loss.

Biomarker	Time Interval	No HBsAg-Loss	Functional Cure	*p* Value
ΔHBsAg (Log IU/mL)	T0–T1	0.02 (−0.02–0.10)	0.19 (−0.04–0.37)	0.082
	T1–T2	0.02 (−0.01–0.09)	0.15 (0–0.27)	0.117
	T0–T2	0.05 (−0.04–0.13)	0.38 (0.11–0.80)	0.002

*p* values were calculated by the Mann-Whitney test. Data are reported as the median and interquartile range. Abbreviations–HBsAg decline (ΔHBsAg), hepatitis B surface antigen (HBsAg), timepoint (T).

## Data Availability

The data presented in this study are available on request from the corresponding author.

## Supplementary Materials

The following are available online at https://www.mdpi.com/article/10.3390/jcm10153308/s1, Table S1: HBsAg values from baseline to last-FU in the overall population and according to functional cure.
