# Supplementary material for: Usefulness of a Hepatitis B Surface Antigen-Based Model for the Prediction of Functional Cure in Patients with Chronic Hepatitis B Virus Infection Treated with Nucleos(t)ide Analogues: A Real-World Study
Source: J Clin Med. 2021 Jul 27;10(15):3308. doi: 10.3390/jcm10153308 (PMC8348455; doi:10.3390/jcm10153308)
Supplement: Supplementary file 1 [file jcm-10-03308-s001.zip › jcm-1288946-supplementary.pdf]

Journal of
Clinical Medicine

Supplementary Materials

Usefulness of a Hepatitis B Surface Antigen-Based Model for the Prediction of Functional Cure in Patients with Chronic Hepatitis B Virus Infection treated with Nucleos(t)ide Analogues: a Real-World Study

Gian Paolo Caviglia, Yulia Troshina, Enrico Garro, Marcantonio Gesualdo, Serena Aneli, Giovanni Birolo, Fabrizia Pittaluga, Rossana Cavallo, Giorgio Maria Saracco and Alessia Ciancio

Table S1. HBsAg values from baseline to the last FU in the overall population and according to functional cure.

Timepoint	Overall Population HBsAg (IU/mL)	No HBsAg Loss HBsAg (IU/mL)	Functional Cure HBsAg (IU/mL)	*p* value
T0	1765 (699–7584)	2236 (812–8911)	13 (3–95)	<0.001
T1	1896 (711–6577)	2357 (793–6784)	6 (1–39)	<0.001
T2	1744 (532–6011)	2362 (866–6582)	4 (1–13)	<0.001
Last-FU	1000 (98–3542)	1472 (428–3880)	0 (0–0)	<0.001

p values were calculated by the Mann-Whitney test. Data are reported as the median and interquartile range. Abbreviations–follow-up (FU), hepatitis B surface antigen (HBsAg), timepoint (T).
